# Endo-lysosomal dysfunction and neuronal–glial crosstalk in Niemann–Pick type C disease

**DOI:** 10.1098/rstb.2022.0388

**Published:** 2024-04-08

**Authors:** Mariagiovanna Malara, Matthias Prestel, Sabina Tahirovic

**Affiliations:** German Center for Neurodegenerative Diseases (DZNE) Munich, 81377 Munich, Germany

**Keywords:** NPC1, cholesterol, endo-lysosomal trafficking, neurons, glia

## Abstract

Niemann–Pick type C (NPC) disease is a rare progressive lysosomal lipid storage disorder that manifests with a heterogeneous spectrum of clinical syndromes, including visceral, neurological and psychiatric symptoms. This monogenetic autosomal recessive disease is largely caused by mutations in the *NPC1* gene, which controls intracellular lipid homeostasis. Vesicle-mediated endo-lysosomal lipid trafficking and non-vesicular lipid exchange via inter-organelle membrane contact sites are both regulated by the NPC1 protein. Loss of NPC1 function therefore triggers intracellular accumulation of diverse lipid species, including cholesterol, glycosphingolipids, sphingomyelin and sphingosine. The NPC1-mediated dysfunction of lipid transport has severe consequences for all brain cells, leading to neurodegeneration. Besides the cell-autonomous contribution of neuronal NPC1, aberrant NPC1 signalling in other brain cells is critical for the pathology. We discuss here the importance of endo-lysosomal dysfunction and a tight crosstalk between neurons, oligodendrocytes, astrocytes and microglia in NPC pathology. We strongly believe that a cell-specific rescue may not be sufficient to counteract the severity of the NPC pathology, but targeting common mechanisms, such as endo-lysosomal and lipid trafficking dysfunction, may ameliorate NPC pathology.

This article is part of a discussion meeting issue ‘Understanding the endo-lysosomal network in neurodegeneration’.

## 1. Niemann–Pick type C disease: a neurovisceral genetic disorder with multisystemic clinical manifestations

**Figure 1.**
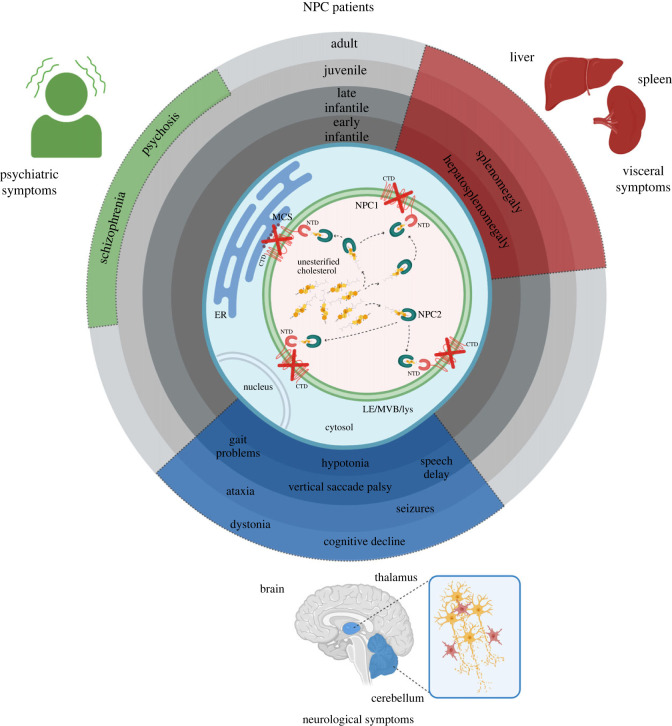
Endo-lysosomal dysfunction causes severe clinical manifestations in Niemann–Pick type C disease. NPC1 (transmembrane protein) and NPC2 (luminal protein) regulate intracellular lipid transport. The hydrophobic handoff model hypothesizes that NPC2 transfers cholesterol to the N-terminal domain of NPC1 [9,10]. Lack of NPC1 or NPC2 impairs the cholesterol egress from the late endo-lysosomal compartment, leading to aberrant lipid storage within the organelles. NPC patients manifest with heterogeneous clinical presentations and disease severity. Dependent on the age of disease manifestation, several forms are acknowledged: early infantile (2 months to <2 years); late infantile (2 to 6 years); juvenile (6 to 15 years) and adult (>15 years). ER: endoplasmic reticulum; MCS: membrane contact sites; LE: late endosome; MVB: multivesicular body; lys: lysosome; NTD: N-terminal domain; CTD: C-terminal domain; NPC1: Niemann-Pick type C intracellular cholesterol transporter 1; NPC2: Niemann-Pick type C intracellular cholesterol transporter 2. Based on reported clinical presentations [3,7]. Created with BioRender.com.

Niemann-Pick type C (NPC) disease is a rare lysosomal lipid storage disorder that manifests with a heterogeneous spectrum of clinical phenotypes, ranging from visceral, to neurological to psychiatric symptoms. Some of the common clinical manifestations of this neurovisceral disorder include hepatosplenomegaly, dystonia, ataxia, epileptic seizures and cognitive decline [1–8] (figure 1). Although first symptoms can be revealed at any age from the neonatal period to the sixth decade of life, the most common manifestation is at childhood age, which often leads to premature death. Notably, the age at neurological onset is largely predictive of disease severity [1,7,11].

Aetiology of this monogenetic disorder is in approximately 95% of the cases attributed to autosomal recessive mutations in the *NPC1* gene, and the remaining 5% of NPC patients carry mutations in the *NPC2* gene [7,12,13]. *NPC1* encodes a large protein of late endosomes/lysosomes (LE/lys) with 13 transmembrane domains that interacts with the small soluble protein encoded by *NPC2* [12,14]. The hydrophobic handoff model hypothesizes that NPC2 transfers cholesterol to the N-terminal domain of NPC1 (figure 1) [9,10]. Their cooperative action ensures that cholesterol and other lipids are exported from the LE/lys, regulating thereby cellular lipid homeostasis [15,16]. The crystal structure of a large fragment of human NPC1 suggests that the ‘sterol-sensing-domain’ shapes a two-way cavity open to both the endosomal lumen and the luminal leaflet of the lipid bilayer [17]. As the structure and size of the cavity may hold a cholesterol molecule, a function of NPC1 in cholesterol sensing and transport has been postulated. In line with these findings, cellular studies showed that mutations in the NPC1 sterol-sensing-domain associate with accumulation of unesterified cholesterol [18]. In addition to the proposed function as a lipid transporter, NPC1 regulates contact sites, facilitating lipid exchange between the organelles [19,20].

## 2. Niemann-Pick type C disease is well recapitulated in rodent models

Mouse models of NPC disease are widely used, recapitulating both the visceral (e.g. hepatosplenomegaly) and neurodegenerative phenotypes [21]. *Npc1*^−/−^ mice with a spontaneous NPC1 mutation (deletion of 11 out of its 13 transmembrane domains) mimic well the human pathology with early disease onset [22–24]. *Npc1*^−/−^ mice initially display a pre-symptomatic phase, followed by mild cerebellar ataxia and tremor at the age of six weeks, which become more prominent at eight weeks of age [21,25]. Severe ataxia, difficulties in food and water uptake, weight loss and premature lethality occur at 10–12 weeks of age. Similar to observations in NPC patients, in mouse models prominent neurodegeneration occurs in cerebellar Purkinje cells and neurons of the thalamus while the cortex and hippocampus appear less affected [8,26,27]. Npc1^spm^ mice, which carry another spontaneous NPC1 mutation, recapitulate similar hallmarks of aggressive NPC pathology [28]. In addition to fast-progressing disease models, NPC pathology is also studied in transgenic mice carrying patient NPC1 mutations such as I1061T, which leads to a partial loss of NPC1 function [29]. This mouse model displays a less aggressive phenotype compared with *Npc1*^−/−^ mice, with the lethality at the age of 15 weeks. Similarly, the Npc1^nmf164^ mouse model, which includes the patient D1005G mutation, recapitulates a more slowly progressing NPC pathology [30].

## 3. A neurovisceral expression of NPC1

NPC1 is expressed throughout the body with high expression in visceral organs, such as adrenal gland, liver, spleen and lungs, as well as in the nervous system (https://www.brainrnaseq.org/). In the nervous system, NPC1 expression is highest in oligodendrocyte precursor cells and myelinating oligodendrocytes, followed by microglia, astrocytes, neurons and endothelial cells (https://www.brainrnaseq.org/). Although the rescue of *Npc1* in visceral organs does restore liver and spleen function, it fails to prevent neurodegeneration and lethality of *Npc1*^−/−^ mice, underscoring the functional importance of NPC1 in the nervous system [31]. In agreement with this, the expression of NPC1 in neurons under the control of the enolase 2 promoter significantly extended the lifespan and ameliorated motor coordination defects [31]. Moreover, the broad expression of NPC1 in the central nervous system, driven by the prion promoter, was sufficient to rescue neurodegeneration and early lethality [32]. Considering the key function of NPC1 in the nervous system and its widespread expression pattern, this review article will assess the cell-autonomous contribution of NPC1 loss in neurons, oligodendrocytes, astrocytes and microglia and discuss the underlying pathomolecular mechanisms mediated by neuronal–glial crosstalk in NPC.

## 4. Impaired lipid trafficking and degradation are key hallmarks of Niemann-Pick type C disease

At the cellular level, defects in NPC1 and NPC2 lead to the accumulation of unesterified cholesterol in LE/lys, and reduced cholesterol levels at the plasma membrane [33,34]. Besides cholesterol, glycosphingolipids, sphingomyelin and sphingosine accumulate in NPC disease, suggesting a broader defect in the homeostasis of diverse lipid species [35–38]. Intriguingly, alterations of the key cellular signalling lipids, namely phosphoinositides, were recently associated with NPC pathology [39]. Differential distribution of phosphoinositide species within the endo-lysosomal pathway is maintained by strict spatio-temporal distribution of their metabolizing enzymes. The resulting phosphoinositide signature of endo-lysosomal compartments is critical for intracellular vesicular trafficking and non-vesicular lipid exchange via membrane contact sites [40]. NPC1 dysfunction leads to alterations in localization of phosphatidylinositol 4-phosphate (PtdIns4P) metabolizing enzymes and, consequently, aberrations in the cellular PtdIns4P gradient. Increased accumulation of PtdIns4P at Golgi and lysosomal membranes causes enhanced anterograde trafficking to the plasma membrane and mechanistic target of rapamycin complex 1 (mTORC1) recruitment to lysosomes, respectively. By controlling the PtdIns4P gradient, NPC1 orchestrates the localization of PtdIns4P-binding proteins shaping the endoplasmic reticulum (ER)–trans-Golgi network and ER–lysosome membrane contact sites [39]. This further supports the key regulatory role of NPC1 in intracellular trafficking and mTORC1 signalling. Notably, NPC1 has been identified as a factor that regulates mTORC1 recruitment and activation in mammalian cell culture systems [41,42]. In turn, active mTORC1 suppresses the initiation of autophagy, connecting mechanistically NPC1 function and autophagy. Previous studies have demonstrated an accumulation of autophagosomes in NPC1-deficient cells [43]. The accumulation of autophagic vesicles can be triggered by a defect in the completion of autophagy and/or enhanced autophagic flux [43–45]. Along these lines, mitophagy—a specific form of autophagy that regulates mitochondrial homeostasis—is aberrant in NPC, contributing to defects in energy supply and metabolism [46,47]. Lysosomal dysfunction and defective mitochondrial turnover are common pathologies observed in lysosomal storage diseases, suggesting a functional relationship between lysosomes and mitochondria [48].

In addition to defective autophagy, impairments along the endo-lysosomal trafficking route were observed upon NPC1 dysfunction, including aberrations in early/recycling endosomes and retromer function [49,50] and accumulation of LE/multivesicular bodies (MVBs) [51,52]. Cholesterol accumulation in MVBs and aberrant delivery to lysosomes in microglia of *Npc1*^−/−^ mice precluded cholesterol export, esterification at the ER and incorporation into lipid droplets [51]. Lack of cholesterol ester-containing lipid droplets in NPC may in turn contribute to metabolic defects and aberrant contact sites as lipid droplets—in addition to their protection against lipotoxicity—represent relevant signalling hubs and organelles for contact site regulation [53]. This is in line with the described role of NPC1 at contact sites that facilitate lipid egress from endocytic organelles to the ER [19]. Taken together, NPC1 is a master regulator of lipid metabolism and endo-lysosomal trafficking, providing rationale for the heterogeneity of cellular phenotypes observed upon its loss.

## 5. The role of NPC1 in brain cholesterol homeostasis

Although 23% of the body cholesterol is found in the brain [54], the entry of cholesterol-rich lipoproteins is prevented by the blood–brain barrier (BBB) [55]. Thus, to maintain the cholesterol homeostasis and membrane integrity in the brain, most of the cholesterol is locally synthesized [56]. Brain cells are particularly vulnerable to impairments in cholesterol handling in NPC, but also in other neurodegenerative diseases [57], supporting the need to better understand how brain cells synthesize and metabolize cholesterol. *In vitro* experiments suggested that the blood–cerebrospinal fluid barrier may serve as a source of brain cholesterol [58]. It has been hypothesized that the apolipoprotein A1-containing high-density lipoprotein (HDL) particles transfer cholesterol from blood capillaries through the choroid plexus epithelium into the cerebrospinal fluid [58–60].

The role of NPC1 in orchestrating cellular cholesterol is well established. However, little is known about the impact of NPC1 loss on cholesterol exchange between neuronal and glial cells. Within the brain parenchyma, the highest proportion of the cholesterol pool is de novo synthesized and provided by astrocytes [55,61,62]. In addition, oligodendrocytes, owing to their especially high demand of cholesterol for myelination in brain development, are capable of synthesizing cholesterol de novo [63]. To the contrary, neurons and microglia rather rely on the uptake of cholesterol [64,65]. For uptake, cholesterol is delivered by the lipoprotein shuttle apolipoprotein E (ApoE) [57,66]. ApoE-containing HDL-like particles—loaded with cholesterol and phospholipids—are secreted by glial cells and the ependymal layer cells [57,67] and endocytosed by the membrane proteins of the low-density lipoprotein receptor (LDLR) gene family [57,66]. Following internalization, LDLRs are recycled to the cell surface and cholesterol cargo is transported to lysosomes and hydrolysed. This unesterified cholesterol is further exported from the lysosome to the ER, where esterification takes place and thereby generated cholesterol esters are stored in lipid droplets. Owing to the major impairment in cholesterol export in NPC, unesterified cholesterol excessively accumulates in LE/MVBs, interrupting thereby the essential lipid uptake and turnover route, with severe consequences for brain cell function [51]. However, it remains to be investigated how neurons or glial cells deal with cholesterol overload in NPC. Excess of cholesterol—especially in neurons—is toxic and tightly regulated under physiological conditions. An important pathway for cholesterol removal is its hydrolysis into the membrane-permeable derivate, 24(*S*)-hydroxycholesterol (24-OHC), executed by the neuronally expressed monooxygenase CYP46A1 [57,68]. 24-OHC is actively exported from the neurons by the ATP-binding cassette transporter A1 (ABCA1) [69] and it is postulated that it crosses the BBB by diffusion [70–72], reaching the circulation, to be catabolized in the liver. Disturbances in levels of 24-OHC were detected in NPC mice and human patients, bringing forward the idea of exploring 24-OHC as a biomarker in NPC clinical trials [73]. As brain glial cells synthesize minor amounts of 24-OHC, it is suggested that the major cholesterol turnover takes place in neurons [74]. To regulate cholesterol homeostasis, 24-OHC also serves as a ligand of the cholesterol sensor liver X receptors (LXRs) [75]. LXRs act corporately as a heterodimer with retinoid X receptor and chromatin remodelling factors, steering the transcriptional programme of trafficking proteins and transporters such as ApoE, ABCA1, ABCG1 [75–77] and likely NPC1 [78]. Additionally, LXRs maintain cholesterol homeostasis by controlling the transcription of the E3 ubiquitin ligase inducible degrader of the LDLR [79], resulting in reduced LDLR levels and, consequently, reduced cellular uptake of cholesterol. An independent pathway for sterol biosynthesis is mediated by the sterol regulatory element binding protein (SREBP). Lack of intracellular cholesterol induces the proteolytic cleavage of SREBP protein and its mature form translocates to the nucleus to regulate the transcription of genes involved in lipid metabolism. Since the *NPC1* gene itself is controlled by the SREBP protein, a feedback inhibition of the SREBP pathway and the LE/lys cholesterol transport by NPC1 may exist [80]. Given the widespread NPC1 expression and aberrations in cholesterol homeostasis observed in different brain cells upon NPC1 dysfunction, we hypothesize that cell-type-specific pathomolecular alterations contribute to NPC disease. Below we discuss our current understanding of brain-cell-specific contributions in NPC by integrating experimental analysis of cell-type restricted depletion of NPC1 and the efficiency of the cell-type-specific rescue of NPC pathology.

## 6. NPC1 function in astrocytes

Astrocytes are an important source of cholesterol for the brain, and astrogliosis is a common hallmark of NPC pathology [81,82]. Depletion of NPC1 in glial fibrillary acidic protein (GFAP)-positive astrocytes, starting at the age of six weeks, was not sufficient to trigger NPC pathology [83]. Along these lines, expression of NPC1 in GFAP-positive astrocytes provided no major benefits to NPC pathology (a trend in increased lifespan and weight gain, no rescue of Purkinje cell neurodegeneration) [31]. By contrast, another study of astrocyte-specific NPC1 rescue using the GFAP promoter reported multiple beneficial effects, including enhanced survival, decreased neuronal cholesterol storage, reduced accumulation of axonal spheroids and lower numbers of degenerated neurons and reactive astrocytes [84]. These discrepancies may be explained by different expression systems that were applied, as Zhang *et al*. [84] used a GFAP promoter fragment and Lopez *et al*. [31] a bi-transgenic/Tet system, which may differentially affect spatial-temporal transgene expression. Intriguingly, NPC1 expression in astrocytes (GFAP promoter) was able to rescue the sterility of *Npc1*^−/−^ mice, but the mechanistic basis of this rescue is still unclear [85]. Direct comparison of the neuronal and astrocytic rescue of NPC pathology revealed an increased survival upon expression of NPC1 in neurons compared with astrocytes, but combining NPC1 expression in astrocytes and neurons had additive effects in prolonging the survival of *Npc1*^−/−^ mice (until the age of 10 months), improving weight loss and delaying ataxia and tremor phenotypes [86]. Taken together, the contribution of astrocytic NPC1 to cholesterol homeostasis in the brain and specific consequences of NPC1 loss in astrocytes for disease pathology need to be further investigated.

## 7. NPC1 function in neuronal cells

Purkinje cell-specific depletion of NPC1 was sufficient to trigger neurodegeneration and motor defects [87]. The cell-autonomous impact of NPC1 loss on Purkinje neurons was demonstrated in chimeric mice, where NPC1-lacking cells degenerated although surrounded by a wild-type environment. The degenerating cells showed an accumulation of autophagic vesicles and MVBs, reflecting defects in intracellular trafficking [88]. However, the specific loss of NPC1 from Purkinje neurons did not induce the weight loss and premature lethality [87] that were observed upon a more global (synapsin 1 promoter) deletion of NPC1 from neurons [83]. Accordingly, Purkinje neuron-specific rescue prevented their degeneration and ameliorated ataxia, but did not prevent the premature lethality of *Npc1*^−/−^ mice [31]. In contrast to beneficial effects observed upon Purkinje neuron-specific rescue, no major improvements were observed upon rescue of NPC1 expression in the forebrain, suggesting differential contribution of various brain regions to the NPC pathology [89]. Besides cell-autonomous phenotypes, neuronal NPC1 also contributes to myelination defects by regulating oligodendrocyte differentiation and maturation [89,90]. The tight interplay between neurons and oligodendrocytes is vital to achieve proper myelination [91], suggesting that multiple cell types are responsible for NPC pathology. Thus, the understanding of the underlying neuronal–glial signalling crosstalk in NPC is of high relevance for designing successful therapies for this complex disease.

## 8. NPC1 function in oligodendrocytes

Cell-specific NPC1 deletion in oligodendrocytes (CNP promoter) revealed a critical role of NPC1 in myelination [89]. Delayed myelination during postnatal development and loss of myelin at later stages were detected upon depletion of NPC1 in oligodendrocytes [89]. It has been hypothesized that the lack of NPC1 protein might be responsible for the diminished expression of the myelin gene regulatory factor, a transcriptional factor essential for oligodendrocyte maturation [92]. This is in agreement with oligodendrocyte maturation defects and transcriptional changes in oligodendrocyte lineage observed upon NPC1 depletion [93,94]. In addition to postulated transcriptional regulation of oligodendrocyte maturation by NPC1, the sequestration of cholesterol in LE/lys is likely to affect myelination [95]. Thus, we postulate that endo-lysosomal dysfunction and autophagy defects in oligodendrocytes are underlying culprits for aberrant myelination in NPC. Phenotypically, loss of NPC1 in oligodendrocytes contributes to motor deficits and ataxia, and even triggers the loss of Purkinje neurons [89]. In this model, Purkinje neurons did not accumulate cholesterol, suggesting that loss of NPC1 function in oligodendrocytes results in non-cell-autonomous degeneration of this vulnerable neuronal population. This example nicely illustrates the complexity of cell-to-cell crosstalk upon endo-lysosomal dysfunction caused by the loss of NPC1. Importantly, demyelination has been reported in a patient presenting with late infantile onset of NPC [96]. As the degree of myelin damage might be connected to the progression of the disease, NPC patients may benefit from monitoring and therapeutic targeting of myelin pathology. However, although oligodendrocyte contribution to aberrant myelination in NPC is well supported, signals provided by neurons and microglia also contribute to myelin pathology in NPC [93] and the mechanistic underpinning of this interaction should be further investigated.

## 9. NPC1 function in microglia

Inflammation and altered innate immune responses are common pathological hallmarks of NPC [25]. NPC1 is highly expressed in microglia [97] and influences their lipid homeostasis and function. Transcriptomic signatures of microglia isolated from symptomatic *Npc1*^−/−^ mice [98] revealed increased disease-associated microglia (DAM) population—defined by Keren-Shaul *et al*. [99]—including upregulation of endo-lysosomal markers, and reduced homeostatic microglial signatures. Transcriptomic changes in symptomatic mice are well aligned with elucidated proteomic signatures of microglia in *Npc1*^−/−^ mice [51] and other neurodegenerative disease models [100]. However, microglial activation is an early pathological manifestation detected in *Npc1*^−/−^ mice, which occurs prior to neurodegeneration and behavioural symptoms [81,82]. Microglia act in concert with neurons to regulate myelin formation by supporting the recruitment of oligodendrocyte progenitor cells (chemoattraction and migration), promoting their proliferation, differentiation/maturation, and clearance of myelin debris [101]. This regulatory role of microglia during early brain development suggests that aberrations in microglial function may have profound consequences for the homeostasis of other brain cells and places microglia in the spotlight of NPC pathology. Noteworthy, loss of NPC1 increases phagocytic activity already during early postnatal stages [51,102] when microglia are involved in shaping of neuronal connectivity. To address early molecular changes in microglia isolated from *Npc1*^−/−^ mice, we analysed their proteomic signatures at postnatal day 7 and found pronounced alterations in endo-lysosomal and autophagy pathways, strongly suggesting early functional engagement of microglia in NPC pathology [51]. At this stage, we also detected impaired lipid homeostasis and lipid droplet formation, caused by accumulation of cholesterol in the LE/MVB compartment. This phenotype, as well as aberrant microglial molecular signatures, was partially rescued by extracting cholesterol from the LE/MVB compartment (using methyl-β-cyclodextrin), connecting mechanistically cholesterol overload and microglial NPC pathology and supporting the idea that excessive lipid storage contributes to microglial dysfunction [51,103]. However, a bona fide cell-autonomous role of NPC1 in microglia was shown by a myeloid-cell-specific depletion of NPC1 (Cx3cr1 promoter) [51]. In contrast to neuronal depletion of NPC1, myeloid depletion did not trigger early lethality. However, depletion of NPC1 in microglia was sufficient to trigger cholesterol accumulation and aberrant endo-lysosomal and autophagy signatures, supporting the relevance of NPC1 signalling for microglial homeostasis [51]. Further analysis of this mouse model is needed as it offers a useful tool to study whether lipid dysfunction in microglia has functional consequences for astrocytes, oligodendrocytes and neurons, and recapitulates NPC pathology.

## 10. Neuronal–glial crosstalk in NPC

**Figure 2.**
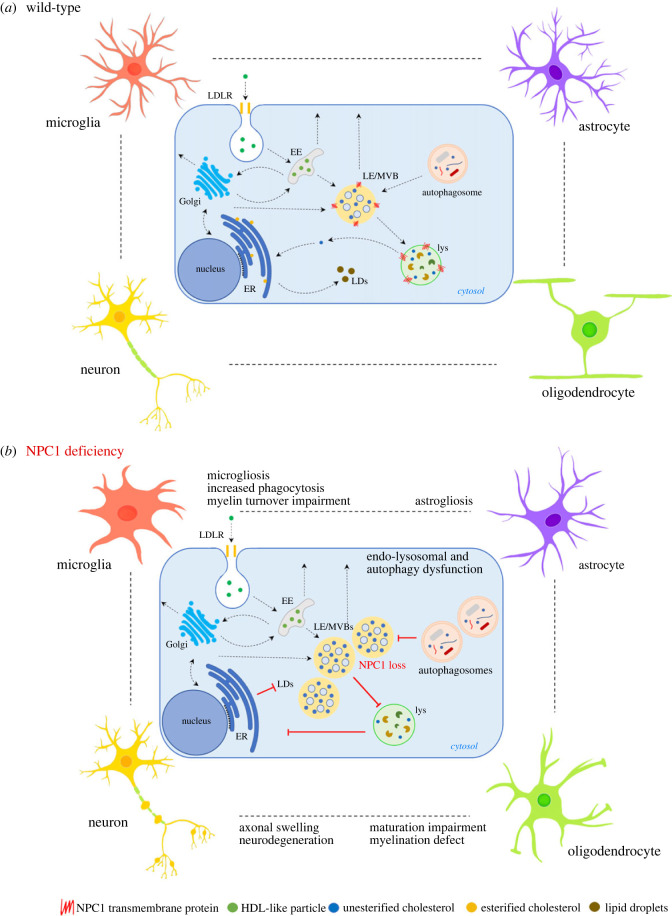
Endo-lysosomal trafficking and neuronal–glial crosstalk in NPC disease. Neuropathology of NPC includes aberrations in endo-lysosomal trafficking and autophagy that have severe functional consequences for brain cell homeostasis, as illustrated by a broad range of pathological hallmarks observed in neurons, oligodendrocytes, microglia and astrocytes. (*a*) Endocytosis of HDL-like particles is mediated by membrane proteins of the LDLR gene family. Following membrane invagination, LDLRs are recycled to the cell surface while lipids, such as cholesterol, are transported to LE/lys for further processing. Unesterified (free) cholesterol is exported from LE/lys to ER, where it is esterified and stored in lipid droplets. (*b*) Loss of NPC1 results in trafficking impairments, leading to accumulation of cholesterol in LE/MVBs, precluding cholesterol export, esterification at the ER and incorporation into lipid droplets. EE: early endosome; LE: late endosome; MVB: multivesicular body; lys: lysosome; ER: endoplasmic reticulum; HDL: high-density lipoprotein; LDLR: low-density lipoprotein receptor; LD: lipid droplet; NPC1: Niemann–Pick type C intracellular cholesterol transporter 1.

Although multiple findings support the idea of a cell-autonomous neurodegeneration, contributions of non-cell autonomous mechanisms to NPC pathology are increasingly recognized. We postulate that understanding of a tight interplay between neurons, oligodendrocytes, astrocytes and microglia is a key to elucidate the pathological complexity of NPC (figure 2). Mechanistic characterization of neuronal–glial interactions has to be better investigated in both rodent and human models of NPC. We believe that a holistic approach to NPC pathology is needed as cell-specific rescue may not be sufficient to stop the aggressive disease pathology. This opinion is supported by above-discussed cell-type-specific rescue experiments. Along these lines, beneficial effects on NPC pathology were observed by combinatorial therapy including lipid reducing and neuroinflammatory strategies [104]. Exploring the neuronal–glial interaction may provide a new perspective for NPC treatment strategies as it may delineate key cellular features that are pre-requisite for the rescue of NPC pathology. We showed that targeting intracellular trafficking to rescue a block of LE/MVB transport to lysosomes may help to reduce lipid burden and microglial pathology. By targeting such core pathological mechanisms, we hopefully can modify NPC pathology across different disease-relevant cells.

## 11. A new perspective to model NPC1-related pathology: from mouse to human cells

Animal models have provided useful mechanistic understanding of the NPC disorder. To translate findings from mouse to humans, human induced pluripotent stem cells (iPSCs)-based technologies are being explored to model the disease. NPC disease is of advantage for iPSC modelling as it is both genetic and developmental, increasing the likelihood to be able to recapitulate disease phenotypes in a dish. As NPC patients present with multiple mutations that mostly occur in combination (compound heterozygous), iPSC-derived models provide valuable tools to study patient NPC mutations and their disease phenotypes such as lipid storage and defects in autophagy [105,106]. It was shown that reprogramming of human fibroblasts isolated from a patient with an early infantile disease onset (compound heterozygous NPC1 mutations c.1628delC/G612D) and differentiated into iPSC-derived neurons recapitulated cholesterol accumulation—the key hallmark of NPC pathology [107]. Cholesterol accumulation was also observed in iPSC-derived neuronal cells of a patient carrying I1061T/P237S NPC1 mutations [108], supporting the robustness of iPSC-derived models in reproducing NPC phenotypes. iPSC-derived cells from a patient with late onset disease, carrying a compound heterozygous NPC1 mutation (p.V1023Sfs*15/p.G992R), were recently characterized [109]. Intriguingly, iPSC-derived neuronal progenitor cells displayed lower levels of NPC1 protein compared with iPSC-derived hepatocyte-like cells or fibroblasts. Although cholesterol accumulation was detected in all three cellular models, iPSC-derived hepatocyte-like cells showed highest cholesterol accumulation, supporting cell-specific differences in cholesterol storage and a role of NPC1 in visceral tissue [109]. Thus, by integrating available patient clinical data and iPSC-derived cellular phenotypes, we can advance the understanding of heterogeneous genetic and biochemical phenotypes in NPC disease [12,110]. The ultimate goal is to understand the pathology of individual NPC mutation carriers [12,13] and develop patient-tailored therapeutic opportunities.

In addition to patient-derived cells, iPSCs were employed to study NPC disease by introducing CRISPR-Cas9-mediated patient mutations. An iPSC-derived neuronal model of NPC revealed accumulation of cholesterol and gangliosides, together with increased lysosomal acidification, mitochondrial defects, and impairments of axonal anterograde and retrograde transport [111]. Characterization by electron microscopy showed multilamellar inclusion bodies, which are often observed in NPC models and illustrate intracellular trafficking defects. Notably, hydroxypropyl-β-cyclodextrin could rescue cholesterol storage, mitochondrial defects and axonal transport [111], supporting the value of iPSC systems for future disease modelling and testing of therapeutic interventions.

Although the above-mentioned models represent an advantageous tool to investigate biochemical phenotypes of NPC, they are not suitable for studies of the intercellular crosstalk. To overcome this obstacle, the intrinsic property of stem cells to spontaneously self-organize in three-dimensional structures offers an advantage. Brain-like regions along the rostro-caudal and dorso-ventral pathways can give rise to so-called cerebral organoids that mimic some aspects of the *in vivo* brain tissue [112]. NPC organoids generated from patient-derived fibroblasts showed reduced proliferation and neuronal differentiation and increased cell death, which were likely contributing to their overall smaller size compared with the wild-type control. Additional pathological changes included cholesterol storage and impaired autophagy [113]. Owing to the inaccessibility of brain tissues from human NPC patients and the efficiency of NPC organoids to recapitulate some of the disease hallmarks, this model system may support studies of NPC pathology and provide a screening tool for therapeutic interventions. However, the lack of microglia or myelinating oligodendrocytes in the organoid model is of disadvantage. The *in vivo* complexity can be mimicked by co-culturing of iPSCs differentiated into neurons, oligodendrocytes, microglia and astrocytes. Individual iPSC cultures need first to be characterized for their pathological hallmarks, by analysing endo-lysosomal trafficking, autophagy, lipid droplet formation, proliferation, cell death, mitochondrial function, lysosomal catalytic activity or lipid storage. Subsequently, cell-type-specific functions, such as axonal and dendritic trafficking, synaptic pruning, metabolic defects, immune function or cholesterol metabolism can be examined in co-culture experiments. Cell-type-specific proteomic profiles or the cellular secretome [114] may elucidate the direct molecular contribution of each cell type to NPC pathology as well as facilitate our understanding of their complex pathological interplay. We believe that mechanistic studies of the neuronal–glial signalling crosstalk in NPC will provide a missing link for designing successful therapies for children suffering from this devastating and incurable disease.

## Data Availability

This article has no additional data.

## References

[1] Evans WR, Hendriksz CJ. 2017 Niemann-Pick type C disease – the tip of the iceberg? A review of neuropsychiatric presentation, diagnosis and treatment. BJPsych Bull. **41**, 109-114. (10.1192/pb.bp.116.054072) 28400970 PMC5376728

[2] Patterson MC et al. 2017 Recommendations for the detection and diagnosis of Niemann-Pick disease type C: an update. Neurol. Clin. Pract. **7**, 499-511. (10.1212/CPJ.0000000000000399) 29431164 PMC5800709

[3] Schneider SA, Tahirovic S, Hardy J, Strupp M, Bremova-Ertl T. 2021 Do heterozygous mutations of Niemann-Pick type C predispose to late-onset neurodegeneration: a review of the literature. J. Neurol. **268**, 2055-2064. (10.1007/s00415-019-09621-5) 31701332

[4] Hammond N, Munkacsi AB, Sturley SL. 2019 The complexity of a monogenic neurodegenerative disease: more than two decades of therapeutic driven research into Niemann-Pick type C disease. Biochim. Biophys. Acta Mol. Cell. Biol. Lipids **1864**, 1109-1123. (10.1016/j.bbalip.2019.04.002) 31002946

[5] Pfrieger FW. 2023 The Niemann-Pick type diseases – a synopsis of inborn errors in sphingolipid and cholesterol metabolism. Prog. Lipid Res. **90**, 101225. (10.1016/j.plipres.2023.101225) 37003582

[6] Wheeler S, Sillence DJ. 2020 Niemann-Pick type C disease: cellular pathology and pharmacotherapy. J. Neurochem. **153**, 674-692. (10.1111/jnc.14895) 31608980

[7] Vanier MT. 2010 Niemann-Pick disease type C. Orphanet J. Rare Dis. **5**, 16. (10.1186/1750-1172-5-16) 20525256 PMC2902432

[8] Fiorenza MT, La Rosa P, Canterini S, Erickson RP. 2023 The cerebellum in Niemann-Pick C1 disease: mouse versus man. Cerebellum **22**, 102-119. (10.1007/s12311-021-01347-3) 35040097 PMC7617266

[9] Kwon HJ, Abi-Mosleh L, Wang ML, Deisenhofer J, Goldstein JL, Brown MS, Infante RE. 2009 Structure of N-terminal domain of NPC1 reveals distinct subdomains for binding and transfer of cholesterol. Cell **137**, 1213-1224. (10.1016/j.cell.2009.03.049) 19563754 PMC2739658

[10] Wang ML, Motamed M, Infante RE, Abi-Mosleh L, Kwon HJ, Brown MS, Goldstein JL. 2010 Identification of surface residues on Niemann-Pick C2 essential for hydrophobic handoff of cholesterol to NPC1 in lysosomes. Cell Metab. **12**, 166-173. (10.1016/j.cmet.2010.05.016) 20674861 PMC3034247

[11] Alobaidy H. 2015 Recent advances in the diagnosis and treatment of Niemann-Pick disease type C in children: a guide to early diagnosis for the general pediatrician. Int. J. Pediatr. **2015**, 816593. (10.1155/2015/816593) 25784942 PMC4345273

[12] Millat G, Marçais C, Tomasetto C, Chikh K, Fensom AH, Harzer K, Wenger DA, Ohno K, Vanier MT. 2001 Niemann-Pick C1 disease: correlations between *NPC1* mutations, levels of NPC1 protein, and phenotypes emphasize the functional significance of the putative sterol-sensing domain and of the cysteine-rich luminal loop. Am. J. Hum. Genet. **68**, 1373-1385. (10.1086/320606) 11333381 PMC1226124

[13] Millat G, Baïlo N, Molinero S, Rodriguez C, Chikh K, Vanier MT. 2005 Niemann–Pick C disease: use of denaturing high performance liquid chromatography for the detection of NPC1 and NPC2 genetic variations and impact on management of patients and families. Mol. Genet. Metab. **86**, 220-232. (10.1016/j.ymgme.2005.07.007) 16126423

[14] Davies JP, Ioannou YA. 2000 Topological analysis of Niemann-Pick C1 protein reveals that the membrane orientation of the putative sterol-sensing domain is identical to those of 3-hydroxy-3-methylglutaryl-CoA reductase and sterol regulatory element binding protein cleavage-activating protein. J. Biol. Chem. **275**, 24 367-24 374. (10.1074/jbc.M002184200) 10821832

[15] Vance JE, Karten B. 2014 Niemann-Pick C disease and mobilization of lysosomal cholesterol by cyclodextrin. J. Lipid Res. **55**, 1609-1621. (10.1194/jlr.R047837) 24664998 PMC4109756

[16] Sleat DE et al. 2004 Genetic evidence for nonredundant functional cooperativity between NPC1 and NPC2 in lipid transport. Proc. Natl Acad. Sci. USA **101**, 5886-5891. (10.1073/pnas.0308456101) 15071184 PMC395893

[17] Li X, Wang J, Coutavas E, Shi H, Hao Q, Blobel G. 2016 Structure of human Niemann–Pick C1 protein. Proc. Natl Acad. Sci. USA **113**, 8212-8217. (10.1073/pnas.1607795113) 27307437 PMC4961162

[18] Millard EE, Gale SE, Dudley N, Zhang J, Schaffer JE, Ory DS. 2005 The sterol-sensing domain of the Niemann-Pick C1 (NPC1) protein regulates trafficking of low density lipoprotein cholesterol. J. Biol. Chem. **280**, 28 581-28 590. (10.1074/jbc.M414024200) 15908696

[19] Höglinger D et al. 2019 NPC1 regulates ER contacts with endocytic organelles to mediate cholesterol egress. Nat. Commun. **10**, 4276. (10.1038/s41467-019-12152-2) 31537798 PMC6753064

[20] Martello A, Platt FM, Eden ER. 2020 Staying in touch with the endocytic network: the importance of contacts for cholesterol transport. Traffic **21**, 354-363. (10.1111/tra.12726) 32129938 PMC8650999

[21] Fog CK, Kirkegaard T. 2019 Animal models for Niemann-Pick type C: implications for drug discovery & development. Expert Opin. Drug Discov. **14**, 499-509. (10.1080/17460441.2019.1588882) 30887840

[22] Loftus SK et al. 1997 Murine model of Niemann-Pick C disease: mutation in a cholesterol homeostasis gene. Science **277**, 232-235. (10.1126/science.277.5323.232) 9211850

[23] Pentchev PG, Gal AE, Booth AD, Omodeo-Sale F, Fours J, Neumeyer BA, Quirk JM, Dawson G, Brady RO. 1980 A lysosomal storage disorder in mice characterized by a dual deficiency of sphingomyelinase and glucocerebrosidase. Biochim. Biophys. Acta **619**, 669-679. (10.1016/0005-2760(80)90116-2) 6257302

[24] Pallottini V, Pfrieger FW. 2020 Understanding and treating Niemann-Pick type Cdisease: models matter. Int. J. Mol. Sci. **21**, 8979. (10.3390/ijms21238979) 33256121 PMC7730076

[25] Platt N, Speak AO, Colaco A, Gray J, Smith DA, Williams IM, Wallom KL, Platt FM. 2016 Immune dysfunction in Niemann-Pick disease type C. J. Neurochem. **136**, 74-80. (10.1111/jnc.13138) 25946402 PMC4833189

[26] Tanaka J, Nakamura H, Miyawaki S. 1988 Cerebellar involvement in murine sphingomyelinosis: a new model of Niemann-Pick disease. J. Neuropathol. Exp. Neurol. **47**, 291-300. (10.1097/00005072-198805000-00008) 3130465

[27] Higashi Y, Murayama S, Pentchev PG, Suzuki K. 1993 Cerebellar degeneration in the Niemann-Pick type C mouse. Acta Neuropathol. **85**, 175-184. (10.1007/BF00227765) 8382896

[28] Miyawaki S, Mitsuoka S, Sakiyama T, Kitagawa T. 1982 Sphingomyelinosis, a new mutation in the mouse: a model of Niemann-Pick disease in humans. J. Hered. **73**, 257-263. (10.1093/oxfordjournals.jhered.a109635) 7202025

[29] Praggastis M et al. 2015 A murine Niemann-Pick C1 I1061T knock-in model recapitulates the pathological features of the most prevalent human disease allele. J. Neurosci. **35**, 8091-8106. (10.1523/JNEUROSCI.4173-14.2015) 26019327 PMC4444535

[30] Maue RA et al. 2012 A novel mouse model of Niemann-Pick type C disease carrying a D1005G-Npc1 mutation comparable to commonly observed human mutations. Hum. Mol. Genet. **21**, 730-750. (10.1093/hmg/ddr505) 22048958 PMC3263988

[31] Lopez ME, Klein AD, Dimbil UJ, Scott MP. 2011 Anatomically defined neuron-based rescue of neurodegenerative Niemann-Pick type C disorder. J. Neurosci. **31**, 4367-4378. (10.1523/JNEUROSCI.5981-10.2011) 21430138 PMC3071647

[32] Loftus SK et al. 2002 Rescue of neurodegeneration in Niemann-Pick C mice by a prion-promoter-driven Npc1 cDNA transgene. Hum. Mol. Genet. **11**, 3107-3114. (10.1093/hmg/11.24.3107) 12417532

[33] Karten B, Vance DE, Campenot RB, Vance JE. 2002 Cholesterol accumulates in cell bodies, but is decreased in distal axons, of Niemann-Pick C1-deficient neurons. J. Neurochem. **83**, 1154-1163. (10.1046/j.1471-4159.2002.01220.x) 12437586

[34] Pentchev P, Brady R, Blanchettemackie E, Vanier M, Carstea E, Parker C, Goldin E, Roff C. 1994 The Niemann-Pick C lesion and its relationship to the intracellular distribution and utilization of LDL cholesterol. Biochim. Biophys. Acta **1225**, 235-243. (10.1016/0925-4439(94)90001-9) 8312368

[35] Lloyd-Evans E et al. 2008 Niemann-Pick disease type C1 is a sphingosine storage disease that causes deregulation of lysosomal calcium. Nat. Med. **14**, 1247-1255. (10.1038/nm.1876) 18953351

[36] Zervas M, Somers KL, Thrall MA, Walkley SU. 2001 Critical role for glycosphingolipids in Niemann-Pick disease type C. Curr. Biol. **11**, 1283-1287. (10.1016/s0960-9822(01)00396-7) 11525744

[37] Vruchte D, Lloyd-Evans E, Veldman RJ, Neville DCA, Dwek RA, Platt FM, Van Blitterswijk WJ, Sillence DJ. 2004 Accumulation of glycosphingolipids in Niemann-Pick C disease disrupts endosomal transport. J. Biol. Chem. **279**, 26 167-26 175. (10.1074/jbc.M311591200) 15078881

[38] Lloyd-Evans E, Platt FM. 2010 Lipids on trial: the search for the offending metabolite in Niemann-Pick type C disease. Traffic **11**, 419-428. (10.1111/j.1600-0854.2010.01032.x) 20059748

[39] Kutchukian C, Vivas O, Casas M, Jones JG, Tiscione SA, Simó S, Ory DS, Dixon RE, Dickson EJ. 2021 NPC1 regulates the distribution of phosphatidylinositol 4-kinases at Golgi and lysosomal membranes. EMBO J.. **40**, e105990. (10.15252/embj.2020105990) 34019311 PMC8246069

[40] Posor Y, Jang W, Haucke V. 2022 Phosphoinositides as membrane organizers. Nat. Rev. Mol. Cell Biol. **23**, 797-816. (10.1038/s41580-022-00490-x) 35589852 PMC9117997

[41] Castellano BM et al. 2017 Lysosomal cholesterol activates mTORC1 via an SLC38A9-Niemann-Pick C1 signaling complex. Science **355**, 1306-1311. (10.1126/science.aag1417) 28336668 PMC5823611

[42] Davis OB, Shin HR, Lim C-Y, Wu EY, Kukurugya M, Maher CF, Perera RM, Ordonez MP, Zoncu R. 2021 NPC1-mTORC1 signaling cuples cholesterol sensing to organelle homeostasis and Is a targetable pathway in Niemann-Pick type C. Dev. Cell **56**, 260-276. (10.1016/j.devcel.2020.11.016) 33308480 PMC8919971

[43] Elrick MJ, Yu T, Chung C, Lieberman AP. 2012 Impaired proteolysis underlies autophagic dysfunction in Niemann-Pick type C disease. Hum. Mol. Genet. **21**, 4876-4887. (10.1093/hmg/dds324) 22872701 PMC3607480

[44] Ordonez MP, Roberts EA, Kidwell CU, Yuan SH, Plaisted WC, Goldstein LSB. 2012 Disruption and therapeutic rescue of autophagy in a human neuronal model of Niemann Pick type C1. Hum. Mol. Genet. **21**, 2651-2662. (10.1093/hmg/dds090) 22437840 PMC3363339

[45] Sarkar S et al. 2013 Impaired autophagy in the lipid-storage disorder Niemann-Pick type C1 disease. Cell Rep. **5**, 1302-1315. (10.1016/j.celrep.2013.10.042) 24290752 PMC3957429

[46] Liedtke M, Volkner C, Hermann A, Frech MJ. 2022 Impact of organelle transport deficits on mitophagy and autophagy in Niemann–Pick disease type C. Cells **11**, 507. (10.3390/cells11030507) 35159316 PMC8833886

[47] Ordonez MP. 2012 Defective mitophagy in human Niemann-Pick type C1 neurons is due to abnormal autophagy activation. Autophagy **8**, 1157-1158. (10.4161/auto.20668) 22647841 PMC3429558

[48] Yambire KF, Fernandez-Mosquera L, Steinfeld R, Mühle C, Ikonen E, Milosevic I, Raimundo N. 2019 Mitochondrial biogenesis is transcriptionally repressed in lysosomal lipid storage diseases. eLife **8**, e39598. (10.7554/eLife.39598) 30775969 PMC6379092

[49] Dominko K et al. 2021 Impaired retromer function in Niemann-Pick type C disease is ddependent on intracellular cholesterol accumulation. Int. J. Mol. Sci. **22**, 13256. (10.3390/ijms222413256) 34948052 PMC8705785

[50] Malnar M, Kosicek M, Lisica A, Posavec M, Krolo A, Njavro J, Omerbasic D, Tahirovic S, Hecimovic S. 2012 Cholesterol-depletion corrects APP and BACE1 misstrafficking in NPC1-deficient cells. Biochim. Biophys. Acta **1822**, 1270-1283. (10.1016/j.bbadis.2012.04.002) 22551668

[51] Colombo A et al. 2021 Loss of NPC1 enhances phagocytic uptake and impairs lipid trafficking in microglia. Nat. Commun. **12**, 1158. (10.1038/s41467-021-21428-5) 33627648 PMC7904859

[52] Liao G, Yao Y, Liu J, Yu Z, Cheung S, Xie A, Liang X, Bi X. 2007 Cholesterol accumulation is associated with lysosomal dysfunction and autophagic stress in Npc1^−/−^ mouse brain. Am. J. Pathol. **171**, 962-975. (10.2353/ajpath.2007.070052) 17631520 PMC1959498

[53] Olzmann JA, Carvalho P. 2019 Dynamics and functions of lipid droplets. Nat. Rev. Mol. Cell Biol. **20**, 137-155. (10.1038/s41580-018-0085-z) 30523332 PMC6746329

[54] Dietschy JM, Turley SD. 2004 Thematic review series: brain lipids. Cholesterol metabolism in the central nervous system during early development and in the mature animal. J. Lipid Res. **45**, 1375-1397. (10.1194/jlr.R400004-JLR200) 15254070

[55] Loving BA, Bruce KD. 2020 Lipid and lipoprotein metabolism in microglia. Front. Physiol. **11**, 393. (10.3389/fphys.2020.00393) 32411016 PMC7198855

[56] Pfrieger FW. 2003 Cholesterol homeostasis and function in neurons of the central nervous system. Cell. Mol. Life Sci. **60**, 1158-1171. (10.1007/s00018-003-3018-7) 12861382 PMC11138592

[57] Zhang J, Liu Q. 2015 Cholesterol metabolism and homeostasis in the brain. Protein Cell **6**, 254-264. (10.1007/s13238-014-0131-3) 25682154 PMC4383754

[58] Fujiyoshi M, Ohtsuki S, Hori S, Tachikawa M, Terasaki T. 2007 24*S*-Hydroxycholesterol induces cholesterol release from choroid plexus epithelial cells in an apical- and apoE isoform-dependent manner concomitantly with the induction of ABCA1 and ABCG1 expression. J. Neurochem. **100**, 968-978. (10.1111/j.1471-4159.2006.04240.x) 17101031

[59] Mahley RW. 2016 Central nervous system lipoproteins: ApoE and regulation of cholesterol metabolism. Arterioscler. Thromb. Vasc. Biol. **36**, 1305-1315. (10.1161/ATVBAHA.116.307023) 27174096 PMC4942259

[60] Pifferi F, Laurent B, Plourde M. 2021 Lipid transport and metabolism at the blood-brain interface: implications in health and disease. Front. Physiol. **12**, 645646. (10.3389/fphys.2021.645646) 33868013 PMC8044814

[61] Ito J, Nagayasu Y, Miura Y, Yokoyama S, Michikawa M. 2014 Astrocyte׳s endogenous apoE generates HDL-like lipoproteins using previously synthesized cholesterol through interaction with ABCA1. Brain Res. **1570**, 1-12. (10.1016/j.brainres.2014.04.037) 24814386

[62] Lindner K et al. 2022 Isoform- and cell-state-specific lipidation of ApoE in astrocytes. Cell Rep. **38**, 110435. (10.1016/j.celrep.2022.110435) 35235798

[63] Marangon D, Boccazzi M, Lecca D, Fumagalli M. 2020 Regulation of oligodendrocyte functions: targeting lipid metabolism and extracellular matrix for myelin repair. J. Clin. Med. **9**, 470. (10.3390/jcm9020470) 32046349 PMC7073561

[64] Martin MG, Pfrieger F, Dotti CG. 2014 Cholesterol in brain disease: sometimes determinant and frequently implicated. EMBO Rep. **15**, 1036-1052. (10.15252/embr.201439225) 25223281 PMC4253844

[65] Pfrieger FW, Ungerer N. 2011 Cholesterol metabolism in neurons and astrocytes. Prog. Lipid Res. **50**, 357-371. (10.1016/j.plipres.2011.06.002) 21741992

[66] Holtzman DM, Herz J, Bu G. 2012 Apolipoprotein E and apolipoprotein E receptors: normal biology and roles in Alzheimer disease. Cold Spring Harb. Perspect. Med. **2**, a006312. (10.1101/cshperspect.a006312) 22393530 PMC3282491

[67] Demattos RB et al. 2001 Purification and characterization of astrocyte-secreted apolipoprotein E and J-containing lipoproteins from wild-type and human apoE transgenic mice. Neurochem. Int. **39**, 415-425. (10.1016/s0197-0186(01)00049-3) 11578777

[68] Lund EG, Guileyardo JM, Russell DW. 1999 cDNA cloning of cholesterol 24-hydroxylase, a mediator of cholesterol homeostasis in the brain. Proc. Natl Acad. Sci. USA **96**, 7238-7243. (10.1073/pnas.96.13.7238) 10377398 PMC22064

[69] Matsuda A, Nagao K, Matsuo M, Kioka N, Ueda K. 2013 24(*S*)-Hydroxycholesterol is actively eliminated from neuronal cells by ABCA1. J. Neurochem. **126**, 93-101. (10.1111/jnc.12275) 23600914

[70] Lütjohann D, Breuer O, Ahlborg G, Nennesmo I, Sidén A, Diczfalusy U, Björkhem I. 1996 Cholesterol homeostasis in human brain: evidence for an age-dependent flux of 24*S*-hydroxycholesterol from the brain into the circulation. Proc. Natl Acad. Sci. USA **93**, 9799-9804. (10.1073/pnas.93.18.9799) 8790411 PMC38509

[71] Bjorkhem I. 2007 Rediscovery of cerebrosterol. Lipids **42**, 5-14. (10.1007/s11745-006-1003-2) 17393206

[72] Björkhem I, Leoni V, Svenningsson P. 2019 On the fluxes of side-chain oxidized oxysterols across blood-brain and blood-CSF barriers and origin of these steroids in CSF (Review). J. Steroid Biochem. Mol. Biol. **188**, 86-89. (10.1016/j.jsbmb.2018.12.009) 30586624

[73] Tortelli B et al. 2014 Cholesterol homeostatic responses provide biomarkers for monitoring treatment for the neurodegenerative disease Niemann-Pick C1 (NPC1). Hum. Mol. Genet. **23**, 6022-6033. (10.1093/hmg/ddu331) 24964810 PMC4204776

[74] Ramirez DM, Andersson S, Russell DW. 2008 Neuronal expression and subcellular localization of cholesterol 24-hydroxylase in the mouse brain. J. Comp. Neurol. **507**, 1676-1693. (10.1002/cne.21605) 18241055 PMC4015140

[75] Courtney R, Landreth GE. 2016 LXR regulation of brain cholesterol: from development to disease. Trends Endocrinol. Metab. **27**, 404-414. (10.1016/j.tem.2016.03.018) 27113081 PMC4986614

[76] Huuskonen J, Vishnu M, Fielding PE, Fielding CJ. 2005 Activation of ATP-binding cassette transporter A1 transcription by chromatin remodeling complex. Arterioscler. Thromb. Vasc. Biol. **25**, 1180-1185. (10.1161/01.ATV.0000163186.58462.c5) 15774904

[77] Lu R, Ito J, Iwamoto N, Nishimaki-Mogami T, Yokoyama S. 2009 FGF-1 induces expression of LXRα and production of 25-hydroxycholesterol to upregulate the apoE gene in rat astrocytes. J. Lipid Res. **50**, 1156-1164. (10.1194/jlr.M800594-JLR200) 19229075 PMC2681397

[78] Rigamonti E et al. 2005 Liver X receptor activation controls intracellular cholesterol trafficking and esterification in human macrophages. Circ. Res. **97**, 682-689. (10.1161/01.RES.0000184678.43488.9f) 16141411

[79] Zhang L, Reue K, Fong LG, Young SG, Tontonoz P. 2012 Feedback regulation of cholesterol uptake by the LXR–IDOL–LDLR axis. Arterioscler. Thromb. Vasc. Biol. **32**, 2541-2546. (10.1161/ATVBAHA.112.250571) 22936343 PMC4280256

[80] Garver WS, Jelinek D, Francis GA, Murphy BD. 2008 The Niemann-Pick C1 gene is downregulated by feedback inhibition of the SREBP pathway in human fibroblasts. J. Lipid Res. **49**, 1090-1102. (10.1194/jlr.M700555-JLR200) 18272927 PMC2311449

[81] Baudry M, Yao Y, Simmons D, Liu J, Bi X. 2003 Postnatal development of inflammation in a murine model of Niemann-Pick type C disease: immunohistochemical observations of microglia and astroglia. Exp. Neurol. **184**, 887-903. (10.1016/S0014-4886(03)00345-5) 14769381

[82] Pressey SN, Smith DA, Wong AM, Platt FM, Cooper JD. 2012 Early glial activation, synaptic changes and axonal pathology in the thalamocortical system of Niemann-Pick type C1 mice. Neurobiol. Dis. **45**, 1086-1100. (10.1016/j.nbd.2011.12.027) 22198570 PMC3657200

[83] Yu T, Shakkottai VG, Chung C, Lieberman AP. 2011 Temporal and cell-specific deletion establishes that neuronal Npc1 deficiency is sufficient to mediate neurodegeneration. Hum. Mol. Genet. **20**, 4440-4451. (10.1093/hmg/ddr372) 21856732 PMC3196892

[84] Zhang M, Strnatka D, Donohue C, Hallows JL, Vincent I, Erickson RP. 2008 Astrocyte-only Npc1 reduces neuronal cholesterol and triples life span of Npc1^–/–^ mice. J. Neurosci. Res. **86**, 2848-2856. (10.1002/jnr.21730) 18500759 PMC2634300

[85] Donohue C, Marion S, Erickson RP. 2009 Expression of Npc1 in glial cells corrects sterility in Npc1^–/–^ mice. J. Appl. Genet. **50**, 385-390. (10.1007/BF03195698) 19875890

[86] Borbon I, Totenhagen J, Fiorenza MT, Canterini S, Ke W, Trouard T, Erickson RP. 2012 Niemann-Pick C1 mice, a model of "juvenile Alzheimer's disease", with normal gene expression in neurons and fibrillary astrocytes show long term survival and delayed neurodegeneration. J. Alzheimers Dis. **30**, 875-887. (10.3233/JAD-2012-120199) 22495346

[87] Elrick MJ, Pacheco CD, Yu T, Dadgar N, Shakkottai VG, Ware C, Paulson HL, Lieberman AP. 2010 Conditional Niemann-Pick C mice demonstrate cell autonomous Purkinje cell neurodegeneration. Hum. Mol. Genet. **19**, 837-847. (10.1093/hmg/ddp552) 20007718 PMC2816612

[88] Ko DC, Milenkovic L, Beier SM, Manuel H, Buchanan JA, Scott MP. 2005 Cell-autonomous death of cerebellar Purkinje neurons with autophagy in Niemann-Pick type C disease. PLoS Genet. **1**, 81-95. (10.1371/journal.pgen.0010007) 16103921 PMC1183526

[89] Yu T, Lieberman AP. 2013 Npc1 acting in neurons and glia is essential for the formation and maintenance of CNS myelin. PLoS Genet. **9**, e1003462. (10.1371/journal.pgen.1003462) 23593041 PMC3623760

[90] Takikita S, Fukuda T, Mohri I, Yagi T, Suzuki K. 2004 Perturbed myelination process of premyelinating oligodendrocyte in Niemann-Pick type C mouse. J. Neuropathol. Exp. Neurol. **63**, 660-673. (10.1093/jnen/63.6.660) 15217094

[91] Duncan GJ, Simkins TJ, Emery B. 2021 Neuron-oligodendrocyte interactions in the structure and integrity of axons. Front. Cell Dev. Biol. **9**, 653101. (10.3389/fcell.2021.653101) 33763430 PMC7982542

[92] Qiao L, Yang E, Luo J, Lin J, Yan X. 2018 Altered myelination in the Niemann-Pick type C1 mutant mouse. Histol. Histopathol. **33**, 1311-1321. (10.14670/HH-18-017) 29956298

[93] Bernardo A, De Nuccio C, Visentin S, Martire A, Minghetti L, Popoli P, Ferrante A. 2021 Myelin defects in Niemann-Pick type C disease: mechanisms and possible therapeutic perspectives. Int. J. Mol. Sci. **22**, 8858. (10.3390/ijms22168858) 34445564 PMC8396228

[94] Kunkel TJ, Townsend A, Sullivan KA, Merlet J, Schuchman EH, Jacobson DA, Lieberman AP. 2023 The cholesterol transporter NPC1 is essential for epigenetic regulation and maturation of oligodendrocyte lineage cells. Nat. Commun. **14**, 3964. (10.1038/s41467-023-39733-6) 37407594 PMC10322873

[95] Cantuti-Castelvetri L et al. 2018 Defective cholesterol clearance limits remyelination in the aged central nervous system. Science **359**, 684-688. (10.1126/science.aan4183) 29301957

[96] Kodachi T, Matsumoto S, Mizuguchi M, Osaka H, Kanai N, Nanba E, Ohno K, Yamagata T. 2017 Severe demyelination in a patient with a late infantile form of Niemann-Pick disease type C. Neuropathology **37**, 426-430. (10.1111/neup.12380) 28387450

[97] Zhang Y et al. 2014 An RNA-sequencing transcriptome and splicing database of glia, neurons, and vascular cells of the cerebral cortex. J. Neurosci. **34**, 11 929-11 947. (10.1523/JNEUROSCI.1860-14.2014) PMC4152602 25186741

[98] Cougnoux A et al. 2018 Microglia activation in Niemann-Pick disease, type C1 is amendable to therapeutic intervention. Hum. Mol. Genet. **27**, 2076-2089. (10.1093/hmg/ddy112) 29617956 PMC5985727

[99] Keren-Shaul H et al. 2017 A unique microglia type associated with restricting development of Alzheimer's disease. Cell **169**, 1276-1290. (10.1016/j.cell.2017.05.018) 28602351

[100] Monasor LS et al. 2020 Fibrillar abeta triggers microglial proteome alterations and dysfunction in Alzheimer mouse models. eLife **9**, e54083. (10.7554/eLife.54083) 32510331 PMC7279888

[101] Miron VE. 2017 Microglia-driven regulation of oligodendrocyte lineage cells, myelination, and remyelination. J. Leukoc. Biol. **101**, 1103-1108. (10.1189/jlb.3RI1116-494R) 28250011

[102] Boyle BR et al. 2020 NPC1 deficiency impairs cerebellar postnatal development of microglia and climbing fiber refinement in a mouse model of Niemann-Pick disease type C. Development **147**, dev189019. (10.1242/dev.189019) 32611604 PMC7420841

[103] Gabandé-Rodríguez E, Pérez-Cañamás A, Soto-Huelin B, Mitroi DN, Sánchez-Redondo S, Martínez-Sáez E, Venero C, Peinado H, Ledesma MD. 2019 Lipid-induced lysosomal damage after demyelination corrupts microglia protective function in lysosomal storage disorders. EMBO J.. **38**, e99553. (10.15252/embj.201899553) 30530526 PMC6331723

[104] Williams IM, Wallom K-L, Smith DA, Al Eisa N, Smith C, Platt FM. 2014 Improved neuroprotection using miglustat, curcumin and ibuprofen as a triple combination therapy in Niemann-Pick disease type C1 mice. Neurobiol. Dis. **67**, 9-17. (10.1016/j.nbd.2014.03.001) 24631719

[105] Lee H et al. 2014 Pathological roles of the VEGF/SphK pathway in Niemann-Pick type C neurons. Nat. Commun. **15**, 5514. (10.1038/ncomms6514) PMC4263144 25417698

[106] Maetzel D, Sarkar S, Wang H, Abi-Mosleh L, Xu P, Cheng AÂW, Gao Q, Mitalipova M, Jaenisch R. 2014 Genetic and chemical correction of cholesterol accumulation and impaired autophagy in hepatic and neural cells derived from Niemann-Pick type C patient-specific iPS cells. Stem Cell Rep. **2**, 866-880. (10.1016/j.stemcr.2014.03.014) PMC4050353 24936472

[107] Trilck M, Hübner R, Seibler P, Klein C, Rolfs A, Frech MJ. 2013 Niemann-Pick type C1 patient-specific induced pluripotent stem cells display disease specific hallmarks. Orphanet J. Rare Dis. **8**, 144. (10.1186/1750-1172-8-144) 24044630 PMC3848807

[108] Yu D et al. 2014 Niemann-Pick Disease type C: induced pluripotent stem cell-derived neuronal cells for modeling neural disease and evaluating drug efficacy. J. Biomol. Screen. **19**, 1164-1173. (10.1177/1087057114537378) 24907126 PMC4529815

[109] Volkner C, Liedtke M, Hermann A, Frech MJ. 2021 Pluripotent stem cells for disease modeling and drug discovery in Niemann-Pick type C1. Int. J. Mol. Sci. **22**, 710. (10.3390/ijms22020710) 33445799 PMC7828283

[110] Brogden G, Shammas H, Walters F, Maalouf K, Das AM, Naim HY, Rizk S. 2020 Different trafficking phenotypes of Niemann-Pick C1 gene mutations correlate with various alterations in lipid storage, membrane composition and miglustat amenability. Int. J. Mol. Sci. **21**, 2101. (10.3390/ijms21062101) 32204338 PMC7139583

[111] Prabhu AV et al. 2021 A human iPSC-derived inducible neuronal model of Niemann-Pick disease, type C1. BMC Biol. **19**, 218. (10.1186/s12915-021-01133-x) 34592985 PMC8485536

[112] Lancaster MA, Knoblich JA. 2014 Generation of cerebral organoids from human pluripotent stem cells. Nat. Protoc. **9**, 2329-2340. (10.1038/nprot.2014.158) 25188634 PMC4160653

[113] Lee S-E, Shin N, Kook MG, Kong D, Kim NG, Choi SW, Kang K-S. 2020 Human iNSC-derived brain organoid model of lysosomal storage disorder in Niemann-Pick disease type C. Cell Death Dis. **11**, 1059. (10.1038/s41419-020-03262-7) 33311479 PMC7733597

[114] Tüshaus J et al. 2020 An optimized quantitative proteomics method establishes the cell type-resolved mouse brain secretome. EMBO J. **39**, e105693. (10.15252/embj.2020105693) 32954517 PMC7560198

